# Deep ultraviolet hydrogel based on 2D cobalt-doped titanate

**DOI:** 10.1038/s41377-022-00991-6

**Published:** 2023-01-01

**Authors:** Youan Xu, Baofu Ding, Ziyang Huang, Lixin Dai, Peng Liu, Bing Li, Wei Cai, Hui-Ming Cheng, Bilu Liu

**Affiliations:** 1 Xi’an Research Institute of High Technology, Xi’an, 710025 China; 2 Shenzhen Geim Graphene Center, Tsinghua-Berkeley Shenzhen Institute and Institute of Materials Research, Tsinghua Shenzhen International Graduate School, Tsinghua University, Shenzhen, 518055 China; 3 Institute of Technology for Carbon Neutrality/Faculty of Materials Science and Engineering, Shenzhen Institute of Advanced Technology, CAS, Shenzhen, 518055 China; 4 Shenyang National Laboratory for Materials Science, Institute of Metal Research, Chinese Academy of Sciences, Shenyang, 110016 China; 5 School of Materials Science and Engineering, University of Science and Technology of China, Shenyang, 110016 China

**Keywords:** Liquid crystals, Magneto-optics

## Abstract

Birefringent optical elements that work in deep ultraviolet (DUV) region become increasingly important these years. However, most of the DUV optical elements have fixed birefringence which is hard to be tuned. Here, we invent a birefringence-tunable optical hydrogel with mechano-birefringence effect in the DUV region, based on two-dimensional (2D) low-cobalt-doped titanate. This 2D oxide material has an optical anisotropy factor of 1.5 × 10^–11^ C^2^ J^−1^ m^−1^, larger than maximum value obtained previously, leading to an extremely large specific magneto-optical Cotton-Mouton coefficient of 3.9 × 10^6^ T^−2^ m^−1^. The extremely large coefficient enables the fabrication of birefringent hydrogel in a small magnetic field with an ultra-low concentration of 2D oxide material. The hydrogel can stably and continuously modulate 303 nm DUV light with large phase tunability by varying the strain (compression or stretching) from 0 to 50%. Our work opens the door to design and fabricate new proof-of-concept DUV birefringence-tunable element, as demonstrated by optical hydrogels capable of DUV modulation by mechanical stimuli.

## Abstract (web summary)

New birefringent element which can stably and continuously modulate DUV light by mechanical stimuli (compressing or stretching).

## Graphical abstract

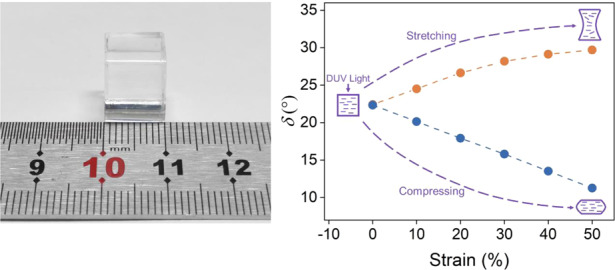

## Introduction

Light modulator provides fine control of phase retardation, intensity, as well as polarization of light, through which information-carrying signals can be superimposed on the electromagnetic waves to realize many uses such as optical communication, digital holography, and adaptive optics^1–4^. Birefringence based light modulator working in the deep ultraviolet (DUV) range (wavelength λ < 350 nm) is highly imperative, as it can finely tune the shape, polarization and phase of DUV pulses without altering the light direction. This feature offers great flexibility for applications in semiconductor processing, optical communication and others fields of medicine and environmental engineering^5–10^. Birefringent element plays the crucial role in determining the performance of the DUV light modulator. Actually, a series of DUV birefringent materials, including single crystals of α-BBO^11^, MgF_2_^12^, Ca(BO_2_)_2_^13^, and α-SnF_2_^14^, have thus been made and commercially used. However, these birefringent elements have the fixed birefringence, limiting their capability of continuous light modulation. Liquid crystals (LCs) are another kind of birefringent materials, of which birefringence is tunable via the molecular alignment by external electrical or magnetic stimuli^15–18^. Up to now, the commonly used LCs are mainly based on organic molecules or polymers, which usually comprise alkyne and contain two different *π* bonds. Such relatively weak bonds are not stable under DUV light due to photochemical degradation effect^19–21^. Meanwhile, DUV can also induce free radicals in some organic groups, and initiate their polymerization, which disorders the alignment and the resultant birefringence of LC^22–25^. Therefore, the birefringent organic LC cannot modulate DUV light.

Thanks to the development of inorganic LCs such as one-dimensional (1D) rod-shape CdSe LC, the fabrication of birefringent elements that can work in DUV region becomes possible due to high stability of these inorganic materials upon DUV exposure^26–29^. Compared to 1D material, two-dimensional (2D) material exhibits larger shape anisotropy (lateral size/thickness ratio is 10^2^–10^5^ for 2D materials while length/diameter ratio is ~10 for 1D CdSe) and the resultant larger optical/magnetic/electrical anisotropy, making it highly sensitive to the external stimulus^30,31^ and exhibiting strong light-matter interaction^32,33^. For instance, we recently discovered a giant magneto-birefringence effect in wide-bandgap 2D materials of cobalt-doped titanium oxide (CTO) and boron nitride, enabling the realization of DUV birefringent element due to the desired DUV stability and removal of DUV-absorptive/instable organic LC and ITO electrodes in the device structure^31,34^. Despite of the development of DUV LC modulator in boron nitride LC, inconvenient magnetic driving way, unclear and uncontrollable magnetic source of 2D boron nitride, as well as difficulty in exfoliating monolayer boron nitride, jointly calling for more research efforts to make this technology practically useful. Noteworthy, 2D CTO holds more promise for controllable DUV modulation as its magnetic source has been confirmed and can be accurately controlled by the molar ratio of Co/Ti in its composition, in addition to the benefit that CTO can be exfoliated in nearly exclusively monolayer forms. However, in addition to the magnetic way, other convenient birefringence-tuning way in DUV region remains elusive, and the dominant factors that determine the giant magneto-birefringence effect of CTO LC is still unclear. Moreover, the molar ratio of Co/Ti is 12% in the previous work, giving rise to the bandgap of 3.4 eV, which blocks the DUV transmittance^31^. In the meantime, hydrogels have attract great attentions, due to their unique properties of precise processability, flexibility, and good compatibility with other active materials^35–37^. Therefore, combination of hydrogel and ultra-wide bandgap 2D material can potentially serve as flexibe DUV optical element with mechanically tunable birefringence.

In this work, by using specially synthesized low-cobalt-doped 2D CTO with a molar ratio of Co/Ti = 6%, the bandgap of the material increases to 3.9 eV, permitting the DUV transmittance and the consequent DUV modulation. Meanwhile, the optical anisotropy factor of 2D CTO is measured to be 1.5 × 10^–11^ C^2^ J^−1^ m^−1^, which is larger than the highest reported value^34^, ensuring its extremely large Cotton-Mouton coefficient. Thanks to the sensitive magnetic response, the birefringent hydrogel is fabricated by crosslinking magnetically aligned 2D materials with the polymer matrix in a small magnetic field range of 0–0.8 T, and an ultra-low concentration of 2D material of 5 × 10^−4^ vol%. The 2D CTO-based hydrogel consequently enables the DUV modulation in a transmissive, mechanical, stable and continuous way, as represented by the one-to-one stretching/compression-phase correspondence.

## Results

We use monolayer CTO as active materials in all following inorganic 2D LC devices. We choose 2D CTO because it is a wide gap semiconductor with high transparency and controllable magnetism^38,39^. The CTO is synthesized by using a wet chemical method (see “Materials and methods” for details). Compared with our previous work^31^, here we used a low Co dopant with a Co/Ti ratio of 6%, to increase the optical bandgap of the materials and ensure a sufficient high transparency at DUV regime. The as-exfoliated 2D CTO flakes exhibit average lateral size and thickness of 1.6 μm and 1.1 nm, and an aspect ratio of ~1500 (Supplementary Fig. S1a–c).

**Fig. 1:**
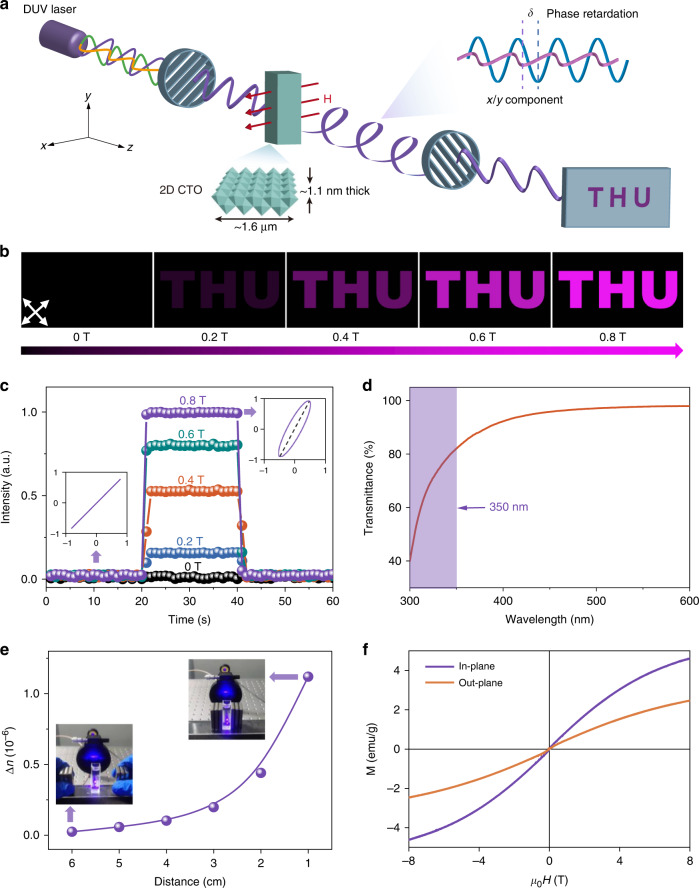
DUV modulation based on 2D CTO LCs with low Co doping. **a** Schematic of the optical setup for magneto-optical measurements. **b** Photographs of the patterned paper precoated with UV-excitable purple phosphor in the magnetic range of 0 T to 0.8 T, with an interval of 0.2 T (white arrow represents the transmission axis of the polarizer). The wavelength of DUV light is set as 303 nm. **c** Intensity of transmitted DUV light versus magnetic field in a forward and reverse scanning. Insets: polarizations of the transmitted DUV light without and with a magnetic field of 0.8 T. **d** Optical transmittance spectrum of non-polarized light through the aqueous suspension of 2D CTO. **e** DUV modulation by controlling the distance between permanent magnets. **f** Magnetization measurements at 10 K in the magnetic field parallel (in-plane) and perpendicular (out-of-plane) to the 2D CTO film produced by vacuum filtration of CTO suspension

Next, we test the DUV modulation capability of the above 2D CTO LC driven by magnetic field. Experimental setup for DUV modulation is shown in Fig. 1a, where a DUV laser with a wavelength of 303 nm is selected as the light source (see “Materials and methods” for details). To visualize the DUV modulation, a colorless paper with a photomask of ‘THU’ pattern and pre-coated UV-excitable phosphor is placed at the rear side of the analyzer. In the absence of magnetic field, no letter is displayed on the paper because two crossed polarizers block the transmittance of DUV light. When the magnetic field is applied and reaches above 0.2 T, the birefringence of CTO suspension is magnetically induced and allowed partial transmission of DUV light, which consequently excites the phosphor to emit visible light and leads to the display of purple ‘THU’ letters. Upon increasing the magnetic field, letters become brighter (Fig. 1b, Supplementary Video 1).

According to Malus’s Law^40^, such field-brightness correspondence is dominated by magnetically tunable phase retardation and the consequent transmitted intensity of DUV light. Replacing the pattern with a photodetector in Fig. 1a, the transmitted intensity is quantitatively monitored, which shows one-to-one correspondence with the strength of magnetic field (Fig. 1c). In addition, the polarization of DUV light is modulated accordingly as evidenced by the polarization evolution from the linear one (0 T) to the elliptical one (0.8 T) due to the magneto-birefringence effect (insets in Fig. 1c). The above results show that the 2D CTO suspension can modulate intensity, phase retardation and polarization of 303 nm DUV light. When a fixed magnetic field is applied, the DUV light can also be manipulated by tuning the angle (*θ*) between magnetic field and the polarization vector of incident light (Supplementary Fig. S1d). Noteworthy, due to its wide optical bandgap of ~3.9 eV (Supplementary Fig. S1e), the 2D CTO aqueous suspension has high optical transparency over the DUV region of 300 nm to 350 nm with an average transmittance of >70%, enabling the DUV modulation in a transmissive way. Compared to conventional reflective modulations, such way need not alter the propagation direction of light, offering greater flexibility.

Besides, owing to the highly sensitive magneto-optic response of our devices, the optical modulation can efficiently work in a low magnetic field. By controlling the distance between permanent magnets, the DUV light is modulated continuously and thus is acceptable for portable devices (Fig. 1e). The sensitive response is closely relative with the intrinsic magnetic anisotropy of 2D CTO, which is characterized by comparing the out-of-plane and in-plane magnetizations of a layered and oriented CTO film fabricated via vacuum filtration. As can be seen from Fig. 1f, CTO material shows the in-plane easy-magnetization axis. The magnetic anisotropy keeps even at room temperature of 300 K, notwithstanding the reduced saturation magnetization compared to that at 10 K due to the thermal break of alignment of magnetic dipoles (Supplementary Fig. S1f).

**Fig. 2:**
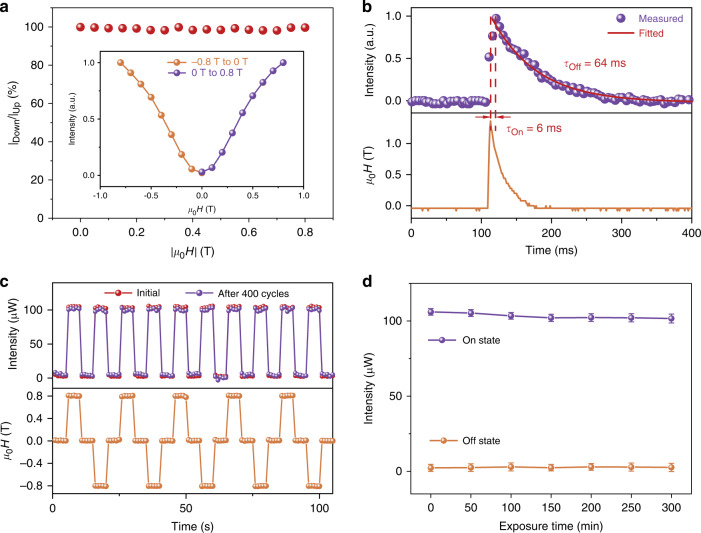
Performance of 2D CTO DUV modulator. **a** Reversibility test of transmitted DUV intensity. Insets: field-intensity correspondence at an interval of 0.1 T from −0.8 T to 0.8 T. **b** Transient magneto-optical signal of transmitted DUV light (upper panel) in response to a magnetic pulse with a peak strength of 1.3 T (lower panel). **c** Cycling test about stability of 2D CTO LC modulator: time-dependent intensity of transmitted DUV light (upper panel) as the magnetic field of ±0.8 T is periodically turned on and off per 10 s (lower panel). **d** Fatigue test of transmitted intensity versus exposure time under continuous DUV irradiation for 300 min. Magnetic field of 0.8 T is kept in turn-on status. DUV light intensity: 200 mW cm^−2^

To characterize performance of the 2D CTO LC DUV modulator, we investigate its reversibility, response time and operation stability. Figure 2a shows the reversibility test of field-intensity correspondence at an interval of 0.1 T from −0.8 T to 0.8 T (opposite magnetic field directions). The intensity of transmitted DUV light is only dependent on the strength of magnetic field rather than its polarity, and no hysteresis is seen between rising process and falling process, confirming the good reversibility. In terms of response time, a transient magneto-optic experiment is carried out using a pulsed magnetic field with an amplitude of 1.3 T and a half width of 7 ms (Fig. 2b). In the rising stage, the magnetic field reaches its peak at 3 ms after the turn-on of pulse, while the peak magneto-optic signal occurs at 9 ms, presenting a fast threshold process of 6 ms. When the field is turned off, intensity undergoes relatively slow decay. Based on the exponential decay function^41^ of $I = I_0e^{ - t/\tau }$, the decay time constant of $\tau$ is fitted to be 64 ms (Fig. 2b), which is almost two orders of magnitude shorter than that of graphene oxide^42^ and hydroxyapatite^43^, permitting fast optical switch. The temperature in this measurement is 300 K. We find that, upon increasing temperature, the rise time and decay time decreases accordingly, due to the decrease of viscosity and the increase of rotational diffusion coefficient of 2D CTO in dispersions^44^ (Supplementary Fig. S2). For the stability, we perform a cycling test by periodically switching on/off the magnetic field of ±0.8 T. Taking a complete on/off operation as a cycle, we observe the negligible degradation (<1%) after 400 cycles, as demonstrated in Fig. 2c. Meanwhile, we also perform the fatigue test, namely, keeping the magnetic field in turn-on status, and exposing CTO LC to DUV light. As illustrated in Fig. 2d, the intensity at working condition (ON state) attenuated slightly (<3%) after continuous DUV exposure for 300 min, further confirming the good DUV stability of 2D CTO LC.

**Fig. 3:**
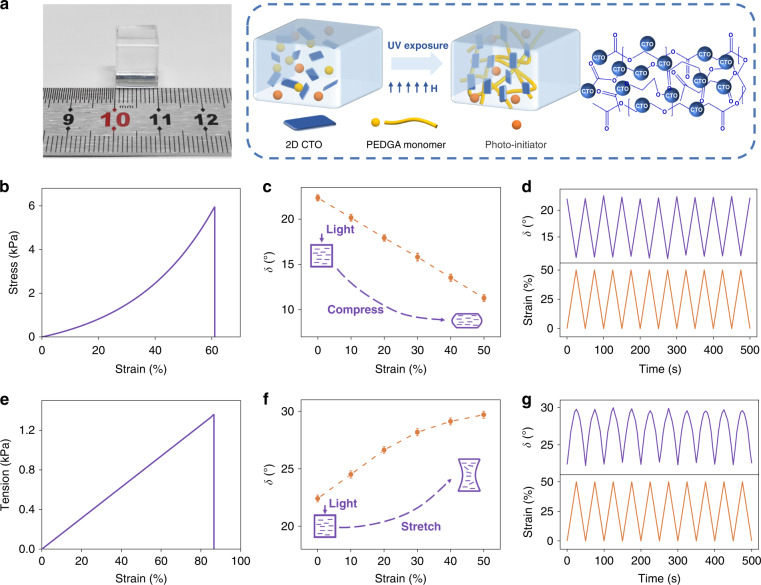
Mechano-optical devices based on 2D CTO DUV hydrogel. **a** A photo of the CTO hydrogel (left) and scheme (right) of its fabrication process. **b** Compressive stress–strain curves of the CTO hydrogel. **c** Phase retardation induced by the uniaxial compression of hydrogel in the direction of light propagation. **d** Cycling test for the reversibility and stability of DUV modulation during compression. **e**–**g** Similar to **b**–**d**, but presenting the process of stretching. DUV light intensity: 200 mW cm^−2^

Noteworthy, the magneto-birefringence effect of 2D CTO LC make it applicable to prepare flexible DUV birefringent optical hydrogel. By adding small amount of monomer and photo-initiator into 2D CTO suspension, we prepare a DUV birefringent hydrogel via in-situ UV curing during exertion of magnetic field (Fig. 3a, Materials and methods, Supplementary Video 2). Once the hydrogelation completed, the magnetically aligned 2D CTO nanosheets maintain inside the hydrogel and all their long axes parallel each other, even after removal of the magnetic field. The CTO hydrogel demonstrates superior flexibility as evidenced by its compression-strain curves (Fig. 3b). In detail, upon applying a slight compression force of 6 kPa, the strain of hydrogel varies from 0% to 60% quickly, with the continuous modulation of DUV light. Phase shift measurements in the light path (parallel to the force) show the linear decrease of phase shift from 23° to 11° with the strain increasing from 0% to 50% (Fig. 3c). Such dependence coincides with the relationship^31,45^ of $\delta = \frac{{2\pi \Delta n_s}}{\lambda }SL$, as the order parameter *S* remains unchanged and length *L* of the optical path through hydrogel is reduced during compression, where $\Delta n_s$ represents saturation birefringence (Supplemental Fig. S3). In addition, the retention rate of hydrogel reaches above 98% after 10 cycles (Fig. 3d), showing the good durability.

Moreover, by exerting the stretch from 0 kPa to 1.3 kPa, the CTO hydrogel deforms with the strain changing from 0% to 50% (Fig. 3e), whereby the phase shift monotonically increased from 22° to 30°, accordingly (Fig. 3f). It is worth noting that besides the increase in *L* along the light path, the stretch also induces the distribution evolution of CTO nanosheets from initial ordered alignment to the disordered one as schemed in Fig. 3f and Supplementary Fig. S3, which nonlinearly decreases the optical anisotropy and the resultant order parameter *S*. Both *S* and *L* vary with the strain, leading to a nonlinear dependence of phase shift with the strain. Durability test also shows the negligible degradation of hydrogel after 10 cycles (Fig. 3g). Based on the above stress–strain-phase correspondence, CTO hydrogel can consequently serve as a transparent mechano-optical crystal, through which the DUV light can be in-situ modulated without direction alteration in a mechanical way. To the best of our knowledge, 2D CTO based hydrogel is the first birefringence-tunable element that can tune the DUV light in a mechanical and continuous way (Supplementary Table. S1).

## Discussion

**Fig. 4:**
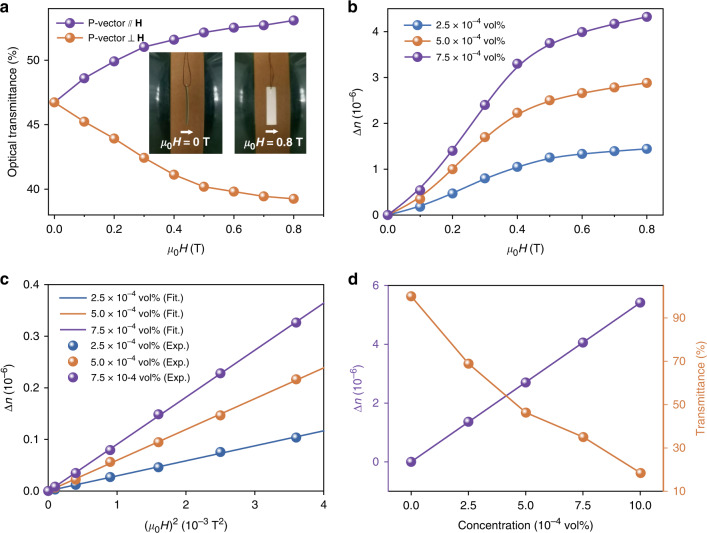
Magneto-birefringence effect of 2D CTO in the DUV region. **a** Optical transmittance of 2D CTO LC (5 × 10^–4^ vol%) in parallel and orthogonal directions of magnetic field to Poynting vector (P-vector). Insets: optical images of a layered and oriented CTO film sample rotated from the initial state (without magnetic field, left) to the direction parallel to the field (0.8 T, right). **b** Magneto-birefringence at different concentrations of CTO. **c** Birefringence as a function of square of magnetic field in the low field range from 0 T to 0.2 T. **d** Concentration dependent birefringence and transmittance of CTO at 0.8 T

We examine the mechanism responsible for the magneto-birefringence of 2D CTO by comparing the transmittance in parallel and orthogonal directions of Poynting vector with respect to external magnetic field (Fig. 4a and Supplementary Fig. S4). At zero field, the CTO flakes orient randomly, exhibiting isotropy with an optical transmittance of 47% at 303 nm. Upon increasing magnetic field, the CTO flakes rotate and align parallel to the field by magnetic torque ${{{\bf{\Gamma }}}} = {{{\bf{M}}}} \times {{{\bf{H}}}}$ due to the magnetic anisotropy^46^, similar to the parallel alignment of layered and oriented CTO film towards the magnetic flux (insets in Fig. 4a). The parallel alignment changes the cross section of 2D materials for DUV scattering, allowing more light to transmit through in parallel direction and blocking the propagation of light in perpendicular direction, which consequently increases (decreases) the transmittance to 53% (39%) (Fig. 4a).

The magneto-birefringence can be calculated from the phase retardation *δ* according to the expression of $\Delta n\left( H \right) = \frac{{\lambda \delta \left( H \right)}}{{2\pi d}}$, where $\delta (H)$ is determined by measuring the magnetic field dependent intensity of transmitted DUV light^47^

1 $$\begin{array}{l} {\delta (H) = m{{{\mathrm{\pi }}}} + 2{{{\mathrm{sin}}}}^{ - 1}\sqrt {\frac{{I - I_{{{{\mathrm{min}}}}}}}{{I_{{{{\mathrm{max}}}}} - I_{{{{\mathrm{min}}}}}}}} \;{{{\mathrm{for}}}}\,m = 0,2,4 \ldots } \\ {\delta (H) = \left( {m + 1} \right){{{\mathrm{\pi }}}} - 2{{{\mathrm{sin}}}}^{ - 1}\sqrt {\frac{{I - I_{{{{\mathrm{min}}}}}}}{{I_{{{{\mathrm{max}}}}} - I_{{{{\mathrm{min}}}}}}}} \;{{{\mathrm{for}}}}\,m = 1,3,5 \ldots } \end{array}$$

where *m* is the number of peaks observed in transmitted intensity, *I* is the intensity at observed state, $I_{{{{\mathrm{max}}}}}$ and $I_{{{{\mathrm{min}}}}}$ are the intensity when the transmission axis of analyzer parallel and perpendicular to that of the polarizer, respectively. Then, the calculated $\Delta n(H)$ is presented in Fig. 4b, which obeys the following equation (Supplementary Note 1)

2 $${{\Delta }}n\left( H \right) = \frac{{\Delta n_{{{{\mathrm{sat}}}}}}}{2}\left[ {3L_2\left( {\frac{{\Delta \chi H^2}}{{2k_BT}}} \right) - 1} \right]$$

where $\Delta n_{{{{\mathrm{sat}}}}}$ is saturation birefringence, $L_2\left( x \right)$ is the 2^nd^ order Langevin function, $\Delta \chi$ is anisotropy of magnetic susceptibility, $k_B$ is Boltzmann constant, and *T* is temperature which is 300 K in following experiments. Similar to electro-optic effect^48^, at the saturation stage, the birefringence can be expressed as $\Delta n\left( H \right) = \Delta n_{{{{\mathrm{sat}}}}} - A\left( {H - H_0} \right)^{ - 2}$, through which saturated birefringence $\Delta n_{{{{\mathrm{sat}}}}}$ is fitted to be 3.2 × 10^–6^ at concentration of 5 × 10^–4^ vol% (Supplementary Fig. S5), where A and $H_0$ are two constants. Thus, the intrinsic optical anisotropy factor $\Delta g$ of 2D CTO is estimated to be 1.5 × 10^–11^ C^2^ J^−1^ m^−1^ utilizing the formula of $\Delta g$ = $\frac{{2n\varepsilon _0}}{\emptyset }\Delta n_{{{{\mathrm{sat}}}}}$, where *n*, *ε*_0_ and $\emptyset$ are average refractive index, vacuum dielectric constant and volume concentration of 2D CTO, respectively. This value is larger than the maximum one in other reported materials^48,49^ (Supplementary Table 2) and thus gives rise to the giant specific Cotton-Mouton coefficient $C_{{{{\mathrm{sp}}}}}$ according to $C_{{{{\mathrm{sp}}}}} = \frac{{\Delta g}}{{2n\varepsilon _0\lambda }}\Delta n_{{{{\mathrm{sat}}}}}\frac{{\partial S}}{{\partial H^2}}$ (Supplementary Note 1), a parameter that describes magnetic sensitivity. At low magnetic field, $C_{{{{\mathrm{sp}}}}}$ is calculated to be 3.9 × 10^6 ^T^−2^ m^−1^ (Fig. 4c). Principally, to achieve the sufficient large birefringence for DUV modulation, a birefringent medium with a larger $C_{{{{\mathrm{sp}}}}}$ requires lower concentration of active material, which will benefit the decrease of viscosity and the increase of DUV transmittance due to the weakened light scattering or absorption (Fig. 4d). Therefore, the obtained giant $C_{{{{\mathrm{sp}}}}}$ makes the 2D CTO LC meet the demands of DUV modulator on both sensitive magneto-response and high optical transmittance for DUV light. Notably, the birefringence monotonically decreases with the temperature. Such temperature dependent behavior originates from the thermally induced Brownian motion of 2D CTO, which tends to disorder the orientations of 2D CTO. (Supplementary Fig. S6).

In summary, we have revealed the dominant role of extremely large optical anisotropy factor of 2D low-cobalt-doped CTO in achieving giant magneto-optic effect in DUV region and invented a DUV hydrogel. The wide bandgap which is induced by low-Co-doping, and large anisotropies in shape, optical factor, and magnetism of 2D CTO collectively endow it with superior capability of DUV modulation, as presented by good reversibility, sensitive magneto-response, and excellent stability. A DUV birefringent crystal with mechano-optical effect has been made by embedding the aligned 2D materials into a hydrogel. Such 2D CTO based inorganic LC and hydrogel can serve as transparent light modulators, featuring DUV modulation capability by either magnetic or mechanical stimulus without changing the optical path. The DUV hydrogel may extend birefringence-tunable optics that are currently used in visible and infrared regions to DUV region, which is important for personalized biomedicine, as well as flexible and soft optical devices.

## Materials and methods

### Synthesis and characterization of low-Co-doped 2D CTO

Suspensions of 2D CTO were prepared by dispersing the as-exfoliated CTO monolayers into water through ultracentrifugation (Optima™ XE-100, Beckman Coulter Life Sciences, USA), where the exfoliation method was similar to the four-stage approach in our previous work^31^ but include an important modification, namely, the content of Co doping was halved because the low content of magnetic element gives a higher optical transparency over the UV spectral range (Supplementary Fig. S7). To be specific, in this work, the Co/Ti ratio was reduced to 6%. Result of the energy dispersive spectrometer (EDS) agrees with stoichiometric ratio of raw materials and gives the atomic ratio of Co: Ti: O = 0.1: 1.69: 4 (Supplementary Fig. S8).

The lateral size and thickness of 2D CTO flakes were characterized using the atomic force microscope (AFM, tapping mode, Cypher ES, Asylum Research, USA). Transmittance spectrum of 2D CTO suspension was measured by UV-vis spectrophotometer (Shimadzu UV-2600, Japan). The high-resolution scanning electron microscopy (SEM, 10 kV, Sigma 300, Carl Zeiss, Germany) equipped with EDS was used to determine the elemental composition. Magnetic anisotropy was determined by using a high-precise magnetometer (Physical Property Measurement System, Quantum Design, USA) to measure the in-plane and out-of-plane magnetizations of a layered and oriented CTO film, which was fabricated by vacuum filtration.

### Optical setup to test performance of 2D CTO DUV modulator

As illustrated in Fig. 1a, a quartz cuvette (1 cm × 1 cm × 5.5 cm) loaded with 2D CTO suspension was placed between two crossed polarizers with a magnetic field applied orthogonal to the optical path. When DUV light propagated through the first polarizer, it was linearly polarized at 45° to the applied magnetic field. Subsequently, the linearly polarized wave was decomposed along two directions, parallel (*x* axis) and orthogonal (*y*-axis) to the external field and subsequently entered the 2D CTO suspension. Nevertheless, due to the magneto-birefringence, two components of linearly polarized wave experienced different refractive index and propagation velocity, giving rise to a phase retardation between them. Thus, the linearly polarized wave was converted into an elliptical wave and was polarized again by the linear analyzer. The magnetic field and light intensity were measured by Hall sensor and spectroradiometer (PR-788, Photo Research, USA), respectively.

### Characterization and fabrication of DUV birefringent hydrogel

A monomer (poly (ethylene glycol) diacrylate, 4 wt%) and a photo-initiator (potassium persulfate, 0.5 wt%) were mixed and dissolved into the aqueous suspension of 2D CTO with a concentration of 5 × 10^–4^ vol%. Subsequently, the mixture was placed into a 1 cm × 1 cm × 1 cm container and then exposed to a UV lamp with the wavelength of 365 nm at 298 K for 10 min. An external magnetic field of 0.8 T was exerted to provide the initial alignment of CTO nanosheets and was then removed after hydrogelation. The stress–strain tests were characterized with a test speed of 2 mm/min (Instron Model 5943, Illinois Tool Works Inc. USA). The DUV modulation performance of the hydrogel was tested based on similar procedure with that of LC devices.

## Supplementary information

Supplementary information for Deep ultraviolet hydrogel based on 2D cobalt-doped titanate

Supplementary Video 1

Supplementary Video 2

## Data Availability

The authors declare that all data supporting the results reported in this study are available within the paper and the Supplementary Information. Additional data used for the study are available from the corresponding author upon reasonable request.

## References

[1] Li SQ (2019). Phase-only transmissive spatial light modulator based on tunable dielectric metasurface. Science.

[2] Shrestha PK, Chun YT, Chu DP (2015). A high-resolution optically addressed spatial light modulator based on ZnO nanoparticles. Light Sci. Appl..

[3] Park J (2021). All-solid-state spatial light modulator with independent phase and amplitude control for three-dimensional LiDAR applications. Nat. Nanotechnol..

[4] Sun ZP, Martinez A, Wang F (2016). Optical modulators with 2D layered materials. Nat. Photonics.

[5] Kneissl M (2019). The emergence and prospects of deep-ultraviolet light-emitting diode technologies. Nat. Photonics.

[6] Xu ZY, Sadler BM (2008). Ultraviolet communications: potential and state-of-the-art. IEEE Commun. Mag..

[7] Park JS (2019). All-glass, large metalens at visible wavelength using deep-ultraviolet projection lithography. Nano Lett..

[8] Oppermann M (2019). Ultrafast broadband circular dichroism in the deep ultraviolet. Optica.

[9] Adamu AI (2019). Deep-UV to Mid-IR supercontinuum generation driven by Mid-IR ultrashort pulses in a gas-filled hollow-core fiber. Sci. Rep..

[10] Wang ZY, Wang XX, Liu JF (2014). Design of nanophotonic, hot-electron solar-blind ultraviolet detectors with a metal-oxide-semiconductor structure. J. Opt..

[11] Zhou GQ (1998). Growth and spectrum of a novel birefringent α-BaB_2_O_4_ crystal. J. Cryst. Growth.

[12] Dodge MJ (1984). Refractive properties of magnesium fluoride. Appl. Opt..

[13] Chen XL (2018). Designing an excellent deep-ultraviolet birefringent material for light polarization. J. Am. Chem. Soc..

[14] Guo JY (2021). α‐SnF_2_: a UV birefringent material with large birefringence and easy crystal growth. Angew. Chem. Int. Ed..

[15] Lin F (2018). Planar alignment of graphene sheets by a rotating magnetic field for full exploitation of graphene as a 2D material. Adv. Funct. Mater..

[16] Wang XZ (2020). A compact full 2π flexoelectro-optic liquid crystal phase modulator. Adv. Mater. Technol..

[17] Coleman DA (2003). Polarization-modulated smectic liquid crystal phases. Science.

[18] Xie ZY (2008). Optical switching of a birefringent photonic crystal. Adv. Mater..

[19] Savage N (2009). Digital spatial light modulators. Nat. Photon..

[20] Kharratian S, Urey H, Onbaşlı MC (2020). Advanced materials and device architectures for magnetooptical spatial light modulators. Adv. Opt. Mater..

[21] Chen R (2016). Improving UV stability of tolane-liquid crystals in photonic applications by the *ortho* fluorine substitution. Opt. Mater. Express.

[22] Lin PT (2004). UV stability of high birefirngence liquid crystals. Mol. Cryst. Liq. Cryst..

[23] Wen CH, Gauza S, Wu ST (2004). Ultraviolet stability of liquid crystals containing cyano and isothiocyanato terminal groups. Liq. Cryst..

[24] Bisoyi HK, Li Q (2016). Light-driven liquid crystalline materials: from photo-induced phase transitions and property modulations to applications. Chem. Rev..

[25] Goossens K (2016). Ionic liquid crystals: versatile materials. Chem. Rev..

[26] Li LS (2002). Semiconductor nanorod liquid crystals. Nano Lett..

[27] Sonin AS (1998). Inorganic lyotropic liquid crystals. J. Mater. Chem..

[28] Miyamoto N, Nakato T (2012). Liquid crystalline inorganic nanosheet colloids derived from layered materials. Isr. J. Chem..

[29] Gabriel JCP (2001). Swollen liquid-crystalline lamellar phase based on extended solid-like sheets. Nature.

[30] Tarafder K (2014). Hole transfer dynamics from a CdSe/CdS quantum rod to a tethered ferrocene derivative. J. Am. Chem. Soc..

[31] Ding BF (2020). Giant magneto-birefringence effect and tuneable colouration of 2D crystal suspensions. Nat. Commun..

[32] Chen HT (2020). All-optical modulation with 2D layered materials: status and prospects. Nanophotonics.

[33] Poh ET, Lim SX, Sow CH (2022). Multifaceted approaches to engineer fluorescence in nanomaterials via a focused laser beam. Light Adv. Manuf..

[34] Xu, H. et al. Magnetically tunable and stable deep-ultraviolet birefringent optics using two-dimensional hexagonal boron nitride. *Nat. Nanotechnol*. 10.1038/s41565-022-01186-1 (2022). 10.1038/s41565-022-01186-1 35953540

[35] Wang Z (2022). Digital holography as metrology tool at micro-nanoscale for soft matter. Light Adv. Manuf..

[36] Yu HY (2021). Three-dimensional direct laser writing of PEGda hydrogel microstructures with low threshold power using a green laser beam. Light Adv. Manuf..

[37] Zhu QL (2020). Distributed electric field induces orientations of nanosheets to prepare hydrogels with elaborate ordered structures and programmed deformations. Adv. Mater..

[38] Xia FN (2014). Two-dimensional material nanophotonics. Nat. Photon..

[39] Osada M (2006). Gigantic magneto–optical effects in multilayer assemblies of two-dimensional titania nanosheets. Adv. Mater..

[40] Wang MS (2014). Magnetically actuated liquid crystals. Nano Lett..

[41] Holzheu S, Hoffmann H (2002). Mechanistic origin of transient electric birefringence anomaly of clay mineral dispersion. J. Phys. Chem. B.

[42] Shen TZ, Hong SH, Song JK (2014). Electro-optical switching of graphene oxide liquid crystals with an extremely large Kerr coefficient. Nat. Mater..

[43] Nakayama M (2018). Stimuli-responsive hydroxyapatite liquid crystal with macroscopically controllable ordering and magneto-optical functions. Nat. Commun..

[44] Loulijat H, Koumina A, Zerradi H (2019). The effect of the thermal vibration of graphene nanosheets on viscosity of nanofluid liquid argon containing graphene nanosheets. J. Mol. Liq..

[45] Dozov I (2011). Electric-field-induced perfect anti-nematic order in isotropic aqueous suspensions of a natural beidellite clay. J. Phys. Chem. B.

[46] Stephen MJ, Straley JP (1974). Physics of liquid crystals. Rev. Mod. Phys..

[47] Pathak G (2018). Analysis of birefringence property of three different nematic liquid crystals dispersed with TiO_2_ nanoparticles. Opto-Electron. Rev..

[48] O’Konski CT, Yoshioka K, Orttung WH (1959). Electric properties of macromolecules. IV. Determination of electric and optical parameters from saturation of electric birefringence in solutions. J. Phys. Chem..

[49] Jennings BR, Wilson SR, Ridler PJ (2005). Magnetic birefringence of minerals. J. Colloid Interface Sci..

