# Supplementary material for: Deep ultraviolet hydrogel based on 2D cobalt-doped titanate
Source: Light Sci Appl. 2023 Jan 1;12:1. doi: 10.1038/s41377-022-00991-6 (PMC9805428; doi:10.1038/s41377-022-00991-6)
Supplement: Supplementary file 1 — Supplementary information for Deep ultraviolet hydrogel based on 2D cobalt-doped titanate [file 41377_2022_991_MOESM1_ESM.docx]

**Supplementary information for**

**Deep ultraviolet hydrogel based on 2D cobalt-doped titanate**

Youan Xu^1,2^, Baofu Ding^2,3^***, Ziyang Huang^2^, Lixin Dai^2^, Peng Liu^4,5^, Bing Li^4,5^, Wei Cai^1^***, Hui-Ming Cheng^2,3,4^, Bilu Liu^2^***

^1^ Xi'an Research Institute of High Technology, Xi’an 710025, China.

^2^ Shenzhen Geim Graphene Center, Tsinghua-Berkeley Shenzhen Institute and Institute of Materials Research, Tsinghua Shenzhen International Graduate School, Tsinghua University, Shenzhen 518055, China.

^3^ Institute of Technology for Carbon Neutrality/Faculty of Materials Science and Engineering, Shenzhen Institute of Advanced Technology, CAS, Shenzhen, 518055, China

^4^ Shenyang National Laboratory for Materials Science, Institute of Metal Research, Chinese Academy of Sciences, Shenyang 110016, China.

^5^ School of Materials Science and Engineering, University of Science and Technology of China, Shenyang 110016, China.

Email: bf.ding@siat.ac.cn; caiwei_bu@163.com; [bilu.liu@sz.tsinghua.edu.cn](mailto:bilu.liu@sz.tsinghua.edu.cn).

**Supplementary Note 1: Model for describing magneto-birefringence effect of 2D CTO.**

We consider each 2D CTO flake in the aqueous as an individual object. In the absence of external magnetic field, all flakes are randomly oriented and the dispersion is optically isotropic. The birefringence can be described as $\Delta n=n_{e}-n_{o}$, where $n_{e}$ (or $n_{o}$) represents the refractive index in the direction parallel (or orthogonal) to magnetic field. Upon increasing the magnetic field, the CTO flakes gradually rotate toward the external field due to the magnetic anisotropy, leading to the ordered alignment. As a result, the anisotropy and birefringence of the 2D CTO dispersion can be induced and expressed by the order parameter *S*, which obeys the formula^1^

$\Delta n=\Delta n_{s\mathrm{at}}S$ (1)

where $\Delta n_{s\mathrm{at}}$ is the saturation birefringence that represents complete orientational ordering. $S$ varies from 0 (isotropic) to 1 (corresponding to $\Delta n_{s\mathrm{at}}$),which is dependent on the magnetic field by the following formula^2,3^

$S(H)=\frac{3}{2}L_{2}(q)-\frac{1}{2}$ (2)

$L_{2}(q)=A^{-1}\int_{0}^{\pi} \cos^{2}\theta exp(q\cos^{2}\theta)sin\thetaⅆ\theta$ (3)

here $A=\int_{0}^{\pi} exp(q\cos^{2}\theta)sin\thetaⅆ\theta$ is the normalized factor, 𝜃 is the angle between the plane of flake and the magnetic field, $L_{2}(q)$ is the generalized second order Langevin function^4^, $q=\Delta\chi H^{2}/2k_{B}T$, where $\Delta\chi$ is anisotropy of magnetic susceptibility, $k_{B}$ is Boltzmann constant, $T$ is temperature. Combining Eq (1)-(3), the birefringence as a function of magnetic field can be written as

$\Delta n\left( H \right)=\frac{\Delta n_{s\mathrm{at}}}{2}\left[ 3L_{2}\left( \frac{\Delta\chi H^{2}}{2k_{B}T} \right)-1 \right]$ (4)

The saturation birefringence can be further described as^5^

${\Delta n}_{\mathrm{sat}}$=$\frac{2n\varepsilon_{0}}{\emptyset}\Delta g$ (5)

where *n*, *ε*_0_, $\emptyset$ and $\Delta g$ are average refractive index, vacuum dielectric constant, volume concentration and optical anisotropy factor of 2D CTO, respectively. Combining Eq (1), (5) and the equation of $C_{sp}=\frac{1}{\emptyset\lambda}\frac{\partial n}{\partial H^{2}}$, the relationship between specific Cotton-Mouton coefficient $C_{sp}$ and $\Delta g$ can be presented as

$C_{sp}=\frac{\Delta g}{2n\varepsilon_{0}\lambda}{\Delta n}_{\mathrm{sat}}\frac{\partial S}{\partial H^{2}}$ (6)

**Supplementary Table 1. Comparison of 2D CTO modulator (this work) with previous reported ones.**

|  | LC-based modulator | External stimuli | Mode*  (T/R) | Ref. |
| --- | --- | --- | --- | --- |
| Commercialized products | PLUTO phase-only | Electric field | R | 6 |
|  | GAEA-2 | Electric field | R | 7 |
|  | ODP512 | Electric field | R | 7 |
|  | X13138 series | Electric field | R | 7 |
|  | X10468 | Electric field | R | 6 |
| R&D  samples | A4907 | Electric field | R | 8 |
|  | Hydroxyapatite | Magnetic field | T | 9 |
|  | RM82 and RM105 stabilized LC | Electric field | T | 10 |
|  | 6 CHBT | Electric field | T | 11,12 |
|  | 2D h-BN | Magnetic field | T | 13 |
| This work | 2D CTO | Mechanical stimuli | T |  |

* Note. “T” represents transmissive and “R” indicates reflective.

**Supplementary Table 2. Comparison of 2D CTO (this work) with other transparent birefringent media in terms of the optical anisotropy.**

| **Materials** | **Optical anisotropy (C^2^ J^-1^ m^-1^)** | **Reference** |
| --- | --- | --- |
| Attapulgite | 3.8×10^-13^ | 5 |
| Bentonite | 3.03×10^-13^ | 5 |
| Montmorillonite | 1.71×10^-13^ | 5 |
| Vermiculite | 1.6×10^-12^ | 5 |
| Hectorite | 4.1×10^-15^ | 5 |
| Kaolinite | 6×10^-14^ | 5 |
| 2D h-BN | 6.5×10^-12^ | 13 |
| 2D CTO | 1.5 × 10^-11^ | This work |

**
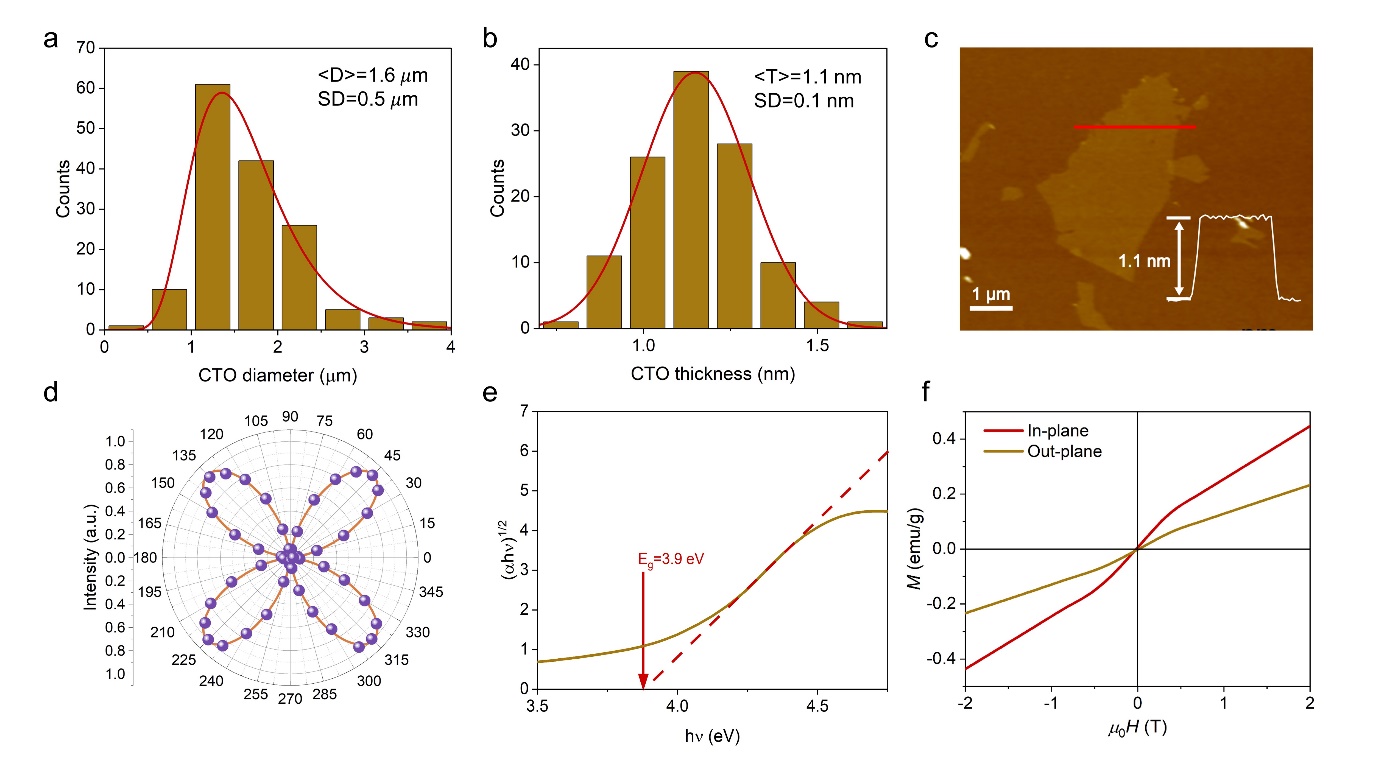
**

**Supplementary Fig. S1 Characterization of low-Co-doped 2D CTO. a, b** Lateral size and thickness distribution of the exfoliated 2D CTO. **c** AFM image of a typical 2D CTO flake. **d** Normalized transmitted intensity with different angle $\theta$ between incident light polarization and magnetic field. **e** Calculation of optical bandgap derived from transmittance spectral based on Tauc’s law. **f** Magnetization measurements at 300 K in parallel (in-plane) and perpendicular (out-of-plane) directions.


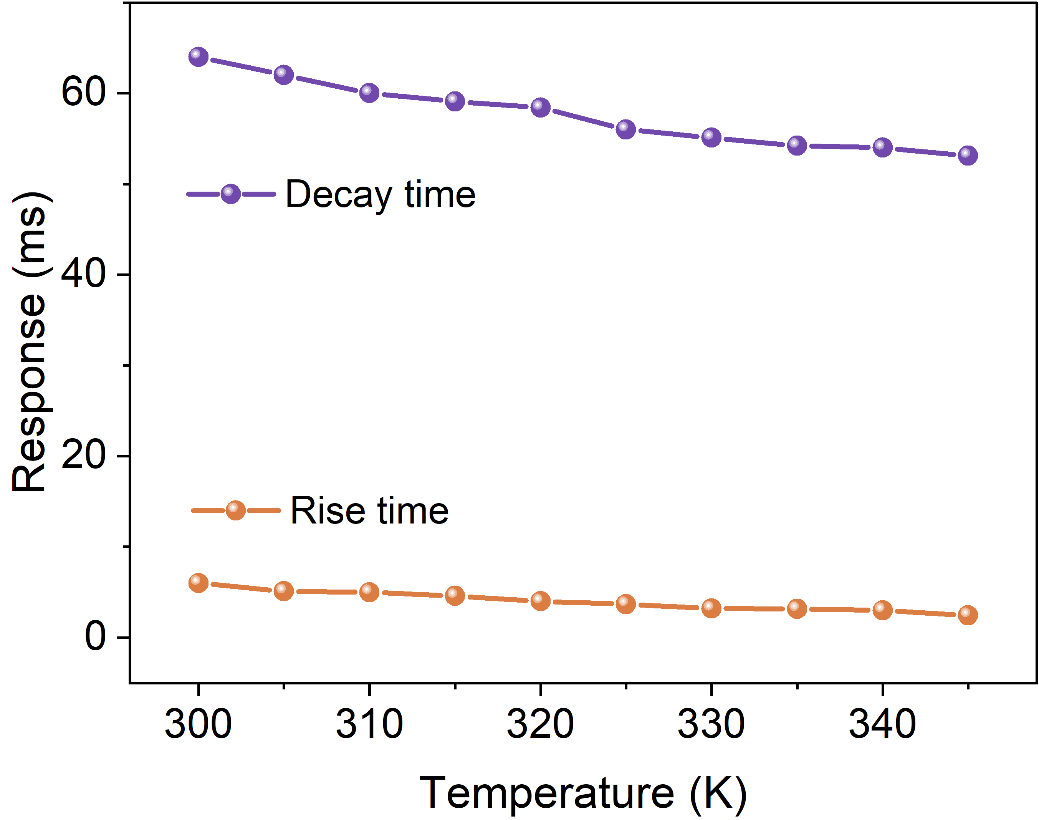


**Supplementary Fig. S2** Temperature dependence of response time of 2D CTO LC.


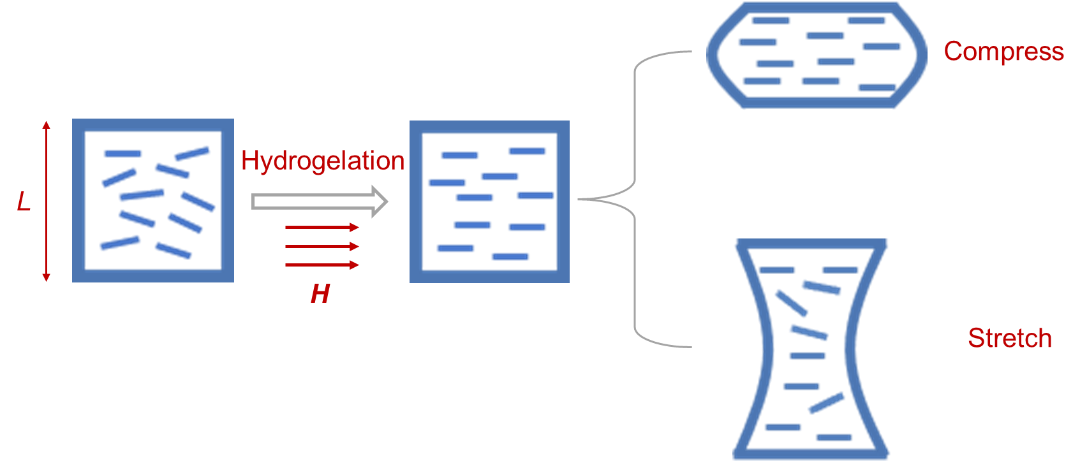


**Supplementary Fig. S3** Schematic diagram of stretching and compression process of 2D CTO DUV hydrogel.


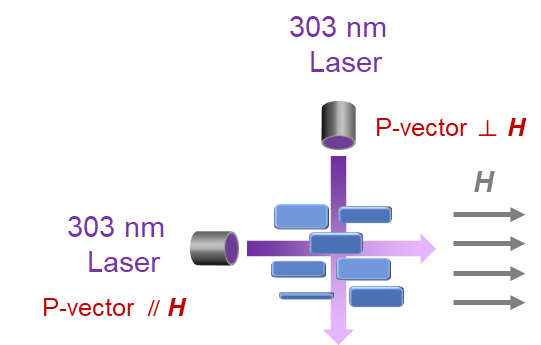


**Supplementary Fig. S4** Optical setup for the transmittance measurement of CTO suspension in two directions.

**Supplementary Fig. S5** Birefringence at as a function of $1/{(\mu_{0}H)}^{2}$, from which the saturated birefringence is fitted to be 3.2 × 10^-6^.


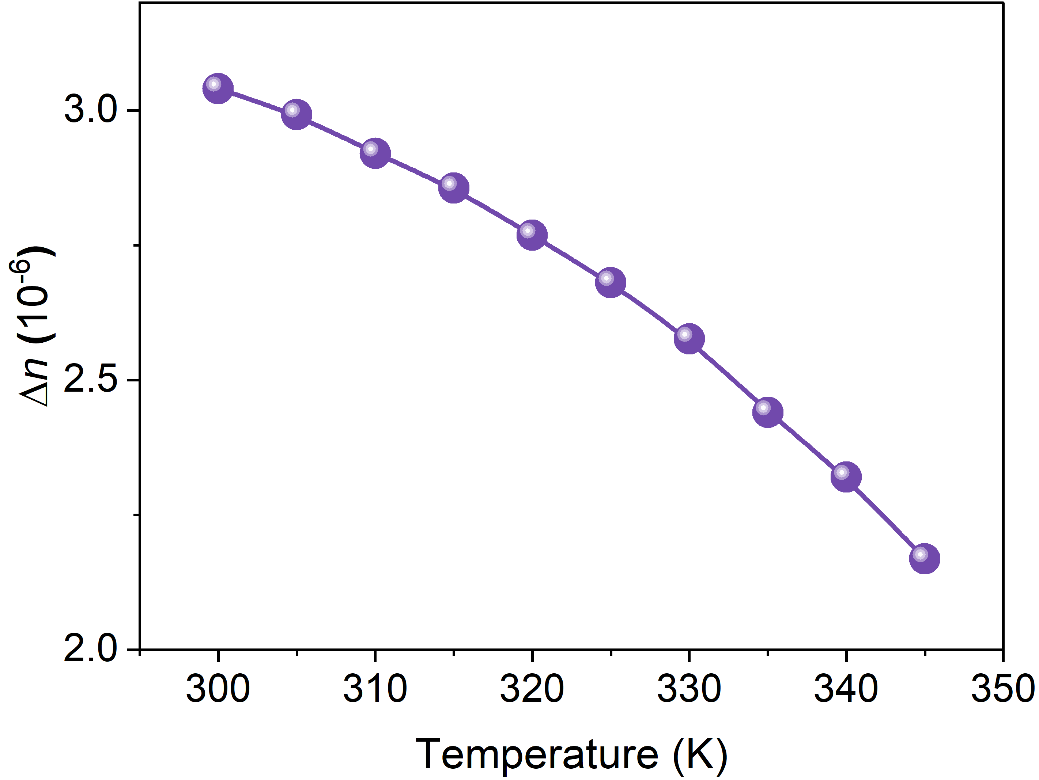


**Supplementary Fig. S6** Temperature dependent magneto-birefringence of 2D CTO LC.

**
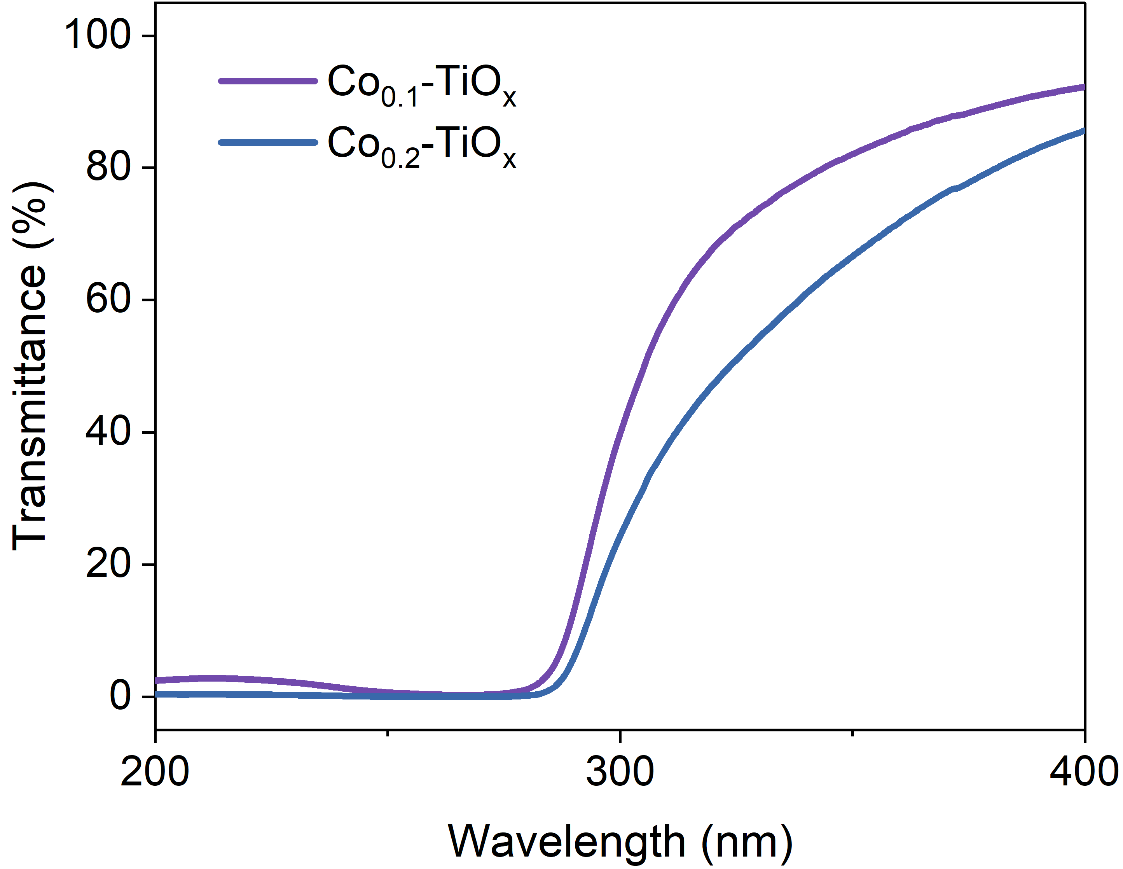
**

**Supplementary Fig. S7** Transmittance of 2D CTO (5 × 10^-4^ vol%) suspension with different contents of Co doping in part of UV spectral region. The reduced content of cobalt (Co_0.1_-TiO_x_ sample) increases the transmittance of DUV light in the range of 300 nm to 350 nm.

**
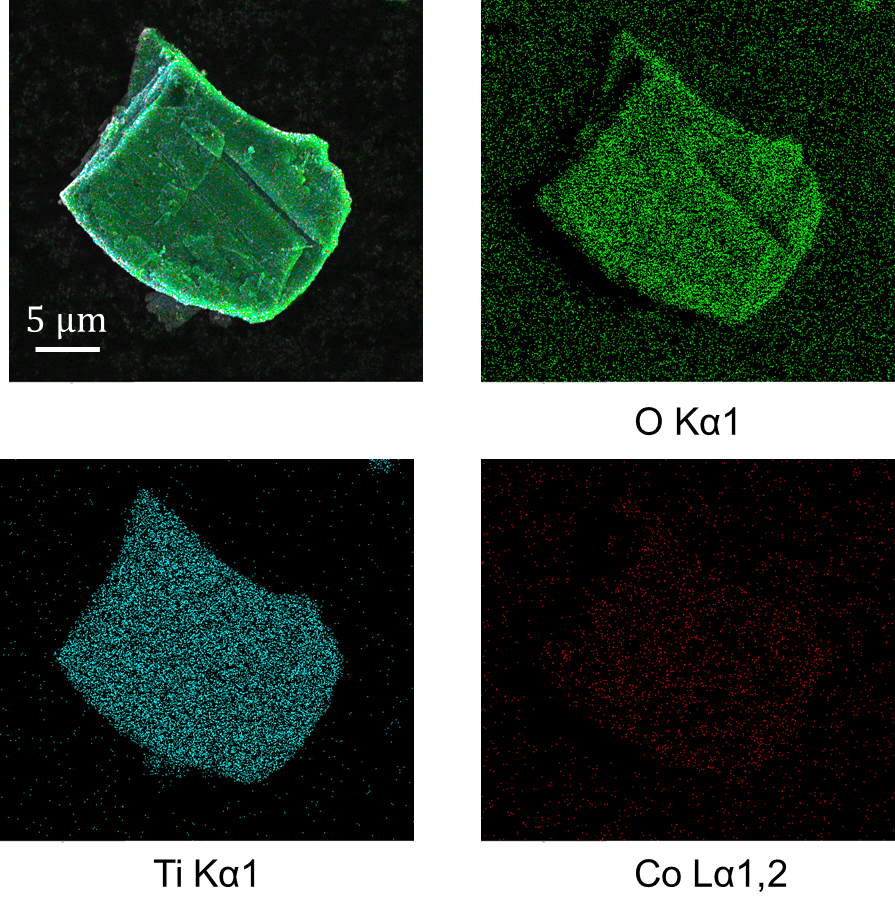
**

**Supplementary Fig. S8** Energy dispersive spectrometer (EDS) mapping of CTO sample. Cobalt is uniformly distributed in the parent layered CTO crystal with the atomic ratio of Co : Ti : O = 0.1 : 1.69 : 4.

**References**

1. Yoshioka, K. & O’konski, C. T. Electric properties of macromolecules. IX. Dipole moment, polarizability, and optical anisotropy factor of collagen in solution from electric birefringence. *Biopolymers* **4**, 499–507 (1966).

2. Stephen, M. J. & Straley, J. P. Physics of liquid crystals. *Rev. Mod. Phys.* **46**, 617–704 (1974).

3. D. Buckingham, A., H. Prichard, W. & H. Whiffen, D. Magnetic birefringence of some diamagnetic gases. *Trans. Faraday Soc.* **63**, 1057–1064 (1967).

4. O’Konski, C. T., Yoshioka, K. & Orttung, W. H. Electric Properties of Macromolecules. IV. Determination of Electric and Optical Parameters from Saturation of Electric Birefringence in Solutions. *J. Phys. Chem.* **63**, 1558–1565 (1959).

5. Jennings, B. R., Wilson, S. R. & Ridler, P. J. Magnetic birefringence of minerals. *J. Colloid Interface Sci.* **281**, 368–376 (2005).

6. Savage, N. Digital spatial light modulators. *Nat. Photonics* **3**, 170–172 (2009).

7. Kharratian, S., Urey, H. & Onbaşlı, M. C. Advanced Materials and Device Architectures for Magnetooptical Spatial Light Modulators. *Adv. Opt. Mater.* **8**, 1901381 (2020).

8. Zou, J. *et al.* Fast-Response Liquid Crystal for Spatial Light Modulator and LiDAR Applications. *Crystals* **11**, 93 (2021).

9. Nakayama, M. *et al.* Stimuli-responsive hydroxyapatite liquid crystal with macroscopically controllable ordering and magneto-optical functions. *Nat. Commun.* **9**, 568 (2018).

10. Zhou, Y. *et al.* Effect of Polymer Network Topology on the Electro-Optical Performance of Polymer Stabilized Liquid Crystal (PSLC) Devices. *Macromol. Chem. Phys.* **221**, 2000185 (2020).

11. Wen, C.-H., Gauza, S. & Wu, S.-T. Ultraviolet stability of liquid crystals containing cyano and isothiocyanato terminal groups. *Liq. Cryst.* **31**, 1479–1485 (2004).

12. Tarjányi, N., Veveričík, M., Káčik, D., Timko, M. & Kopčanský, P. Birefringence dispersion of 6CHBT liquid crystal determined in VIS-NIR spectral range. *Appl. Surf. Sci.* **542**, 148525 (2021).

13. Xu, H. *et al.* Magnetically tunable and stable deep-ultraviolet birefringent optics using two-dimensional hexagonal boron nitride. *Nat. Nanotechnol.* (2022) doi:10.1038/s41565-022-01186-1.
